# Inhibition of soluble epoxide hydrolase by phytochemical constituents of the root bark of *Ulmus davidiana var. japonica*

**DOI:** 10.1080/14756366.2021.1927005

**Published:** 2021-05-17

**Authors:** Jang Hoon Kim, Ji Su Park, Yun Ji Lee, Sena Choi, Young Ho Kim, Seo Young Yang

**Affiliations:** a Department of Herbal Crop Research, National Institute of Horticultural & Herbal Science, RDA, Jeonju, Korea; b College of Pharmacy, Chungnam National University, Daejeon, Republic of Korea; c Department of Pharmaceutical Engineering, Sangji University, Wonju-si, Republic of Korea

**Keywords:** Soluble epoxide hydrolase, *Ulmus davidiana*, *Ulmaceae*, inhibitor, molecular simulation

## Abstract

A novel compound **1** and nine known compounds (**2**–**10**) were isolated by open column chromatography analysis of the root bark of *Ulmus davidiana*. Pure compounds (**1**–**10)** were tested *in vitro* to determine the inhibitory activity of the catalytic reaction of soluble epoxide hydrolase (sEH). Compounds **1**, **2**, **4**, **6**–**8**, and **10** had IC_50_ values ranging from 11.4 ± 2.3 to 36.9 ± 2.6 μM. We used molecular docking to simulate inhibitor binding of each compound and estimated the binding pose of the catalytic site of sEH. From this analysis, the compound **2** was revealed to be a potential inhibitor of sEH *in vitro* and *in silico*. Additionally, molecular dynamics (MD) study was performed to find detailed interaction signals of inhibitor **2** with enzyme. Finally, compound **2** is promising candidates for the development of a new sEH inhibitor from natural plants.

## Introduction

Soluble epoxide hydrolase (sEH, E.C. 3.3.2.10) is a member of the α/β hydrolase family found in both the cytosolic and peroxisomal compartments of the cell. sEH is composed of two independently folding domains within the C-terminal and N-terminal^1^. The C-terminal domain has epoxide hydrolase activity that converts epoxyeicosatrienoic acids (EETs) into dihydroxyeicosatrienoic acids (DHETs), while the N-terminal domain has a phosphatase activity that hydrolases lipid phosphates^2^. EETs derived from arachidonic acid exist in four regioisomers distinguished by the location of epoxide, denoted 5,6-EET, 8,9-EET, 11,12-EET, and 14,15-EET^3^. EETs are secreted into vascular endothelial and renal epithelial cells, where they contribute to amelioration of hypertension and chronic kidney disease as endothelial-derived hyperpolarising factors, and by inhibiting epithelial sodium channels in the kidney^4^.

Additionally, EETs have been shown to suppress vascular inflammation by controlling the phosphor-IkB kinase activity induced by nuclear factor-kB activation^4^^,^^5^. Recently, carbamate urea sEH inhibitors have been used to treat renal injury and decrease blood pressure in animal models^6^. Therefore, sEH inhibitor is considered a powerful tool to treat cardiovascular and inflammatory diseases^7^.

*Ulmus davidiana* var. *japonica* (*U. davidiana*) is a Japanese elm belonging to the Ulmaceae family found in large parts of North-East Asia^8^. The root bark of *U. davidiana*, known as yugeunpi in Korean^9^, has been used both as a tea and an ingredient in foods, such as a thickener for soups and a cereal flour additive^10^. *U. davidiana* is a traditional Korean medicine that has been used for the treatment of inflammation, edoema, cancer, rheumatoid arthritis, haemorrhoids, and mastitis^8^^,^^10^. Previous studies of its biological properties reported that it has anti-oxidant, anti-cancer, anti-inflammatory, and anti-bacterial properties^9–11^. Previous phytochemical studies demonstrated that *U. davidiana* contains various chemical compounds, including phenolic compounds, lignans, and catechins^9^^,^^10^.

The aim of this study is to evaluate the sEH biological activity of components of the root bark of *U. davidiana*. A new compound (**1**) and nine known components (**2**–**10**) were isolated via methanol extraction followed by column chromatography. Structures were elucidated using one- and two-dimensional nuclear magnetic resonance (NMR) and high-resolution electrospray ionisation mass spectrometry (HR-ESI-MS). Finally, we tested the inhibitory activity of each compound on sEH through *in vitro* and *in silico* evaluations.

## Materials and methods

### General experimental procedures

NMR experiments were conducted on an ECA500 instrument (JEOL, Tokyo, Japan), with the chemical shift referenced to the residual solvent signals, and using methanol-*d*_4_ and DMSO-d_6_ as the solvent. Thin-layer chromatography (TLC) analysis was performed on silica-gel 60 F254 and RP-18 F254S plates (both 0.25 mm layer thickness; Merck, Darmstadt, Germany). Compounds were visualised by dipping plates into 10% (v/v) H_2_SO_4_ reagent, which were then air heat-treated at 300 °C for 15 s. Silica gel (60 A, 70–230 or 230–400 mesh ASTM; Merck) and reversed-phase silica gel (ODS-A 12 nm S-150, S-75 μm; YMC Co., Kyoto, Japan) were used for open column chromatography. sEH (10011669), AUDA (479413–70-2), and PHOME (10009134) were purchased from Cayman (Ann Arbour, Michigan, MO).

### Plant material

The root bark of *U. davidiana* was purchased from a herbal company, Republic of Korea, in February 2017. This plant was identified by Prof. Y.H. Kim. A voucher specimen has been deposited in the herbarium of the College of Pharmacy, Chungnam National University, Daejeon, Republic of Korea.

### Extraction and isolation

The dried powder (3 kg) of the root bark of *U. davidiana* was extracted with 70% methanol/30% water (7 L × 3) at ∼55 °C for 3 h. Extraction was repeated four times. Concentrated methanol extract (399.6 g) was suspended in distilled water and progressively fractionated with *n*-hexane (16.9 g), ethyl acetate (E; 41.5 g) and water (409.0 g). The E fraction was subjected to silica gel column chromatography using a gradient solvent system of chloroform and methanol (from 50:1 to 2:1) to obtain seven fractions (E1–7). The E3 fraction was chromatographed by silica gel column chromatography with a gradient solvent system of chloroform and methanol (from 15:1 to 5:1) to obtain three fractions (E31–33). Compounds **9** and **10** were purified by Sephadex LH-20 with mixed solvent system (methanol:water/1:1) from the E32 fraction. The E4 fraction was separated by RP-C18 column chromatography with a gradient solvent system of methanol and water (from 1:2 to 3:1) to obtain five fractions (E41–45). The E41 fraction was subjected to silica gel chromatography using a gradient solvent system of chloroform and methanol (from 10:1 to 6:1) to obtain two fractions (E411 and E412). Two compounds (**4** and **5**) were separated from the E411 fraction by Sephadex LH-20 column chromatography with an isocratic solvent system of chloroform, methanol, and water (7:1:0.1). Compounds **3** and **6** were isolated from the E412 fraction with Sephadex LH-20 column chromatography using an isocratic solvent system of chloroform, methanol and water (7:1:0.1). The E42 fraction was separated by Sephadex LH-20 column chromatography with an isocratic solvent system of methanol and water (2:3) to obtain isolate compound **8**. The E43 fraction was purified by Sephadex LH-20 column chromatography with an isocratic solvent system (methanol:water/1:1) to isolate compound **7**. Compounds **1** and **2** were separated from the E44 fraction with Sephadex LH-20 column chromatography using an isocratic solvent system (methanol:water/1:1).

### sEH inhibition assay

The sEH assay was performed as described previously, with minor modifications^12^. For determining inhibitory activity, 130 µL of ∼83 µg/mL sEH in 25 mM bis-Tris–HCl buffer (pH 7.0) containing 0.1% BSA was added to either 20 µL of inhibitor dissolved in MeOH, or MeOH. Next, 50 µL of the 10 µM substrate (PHOME) was added to each mixture and incubated at 37 °C to allow for sEH hydrolysis. The products were monitored at 330 nm excitation and 465 nm emission for approximately 40 min.

Inhibitory activity was calculated using the following equations:

(1) Inhibitoryactivityrate(%)=[(ΔC–ΔI)/ΔC]×100

where *Δ*C and *Δ*I are the intensity of the control and inhibitor, respectively, after 40 min.

(2) $$y=y_{0}+\left(a\times x/b+x\right)$$

where y_0_ is the minimum value of the *y*-axis, a is the difference between the maximum and minimum values, and b is the *x* value at 50% of the a value.

### Molecular docking

For docking the ligand into the active site of enzyme, two ligands with a 3D structure were constructed and minimised using Chem3D Pro (CambridgeSoft, Cambridge, MA). The protein structure of the enzyme was coded in 3ANS and downloaded from the RCSB protein data bank. Only the A-chain of this enzyme was necessary for docking, so the B-chain was not included. Water and 4-cyano-N-[(1S,2R)-2-phenylcyclopropyl]-benzamide were then excluded from the A-chain. The revised A-chain was added to hydrogen using AutoDockTools (Scripps Research, La Jolla, CA); the Gasteiger charge model was then applied. Flexible ligand docking was achieved using a torsion tree, with detection of the torsion root and rotatable bonds. The grid box was set to a size of 55 × 55 × 55 at 0.375 Å for the docking the ligand into the active site. Molecular docking was achieved via a Lamarckian genetic algorithm with the maximum number of evaluations. The resulting values were calculated and represented using AutoDockTools (La Jolla, CA), Chimaera 1.14 (San Francisco, CA), and LIGPLOT (European Bioinformatics Institute, Hinxton, UK).

### Molecular dynamics

Molecular dynamics (MD) was performed using the Gromacs 4.6.5 package. The 3D structure of ligand was built the GlycoBioChem server. sEH Gro was produced with GROMOS96 53a3 force field from pdb. Their complex was surrounded by water molecules with six Cl anions. The energy minimisation was stabilised up to 10.0 kJ/mol in steepest descent minimisation. The inhibitor **2**-sEH complex was sequentially performed to NVT equilibration at 300K, NPT with Particle Mesh Ewald for long-range electrostatics at 1 bar and MD simulation for 20 ns, respectively.

### Statistical analysis

All measurements were performed in triplicate across three independent experiments, and the results are shown as mean ± standard error of the mean (SEM). The results were analysed using Sigma Plot (Systat Software Inc., San Jose, CS).

## Results and discussion

### Isolation and identification of compounds from the root bark of *U. davidiana*

**Figure 1.**
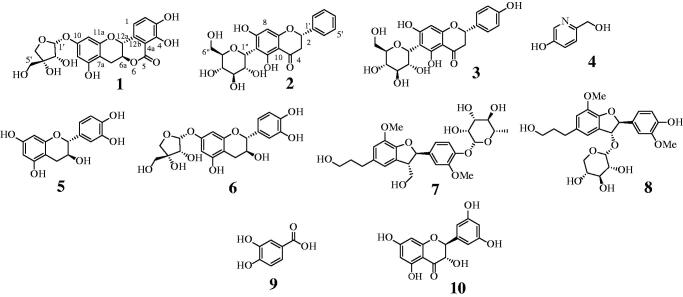
Structures of isolated compounds **1**–**10**.

Recent studies analysing phenolic compounds and flavonoids in the root back of *U. davidiana*^9^^,^^10^ have shown that they have antioxidant^13^ and antibacterial^9^ properties. The methanol extracts of the root bark of *U. davidiana* were sequentially divided into *n*-hexane, ethyl acetate, and water fractions for analysis. The ethyl acetate fraction was separated with open column chromatography, leading to the isolation of a new compound (**1)**, compound (**2**) reported for the first time in natural plant, and eight known compounds (**3–10**): naringenin-6-C-*β*-d-glucopyranoside (**3**)^14^, 6-hydroxymethyl-3-pyridinol (**4**)^15^, (+)-catechin (**5**)^16^, catechin-7-*O*-*β*-apiofuranoside (**6**)^17^, icariside E_4_ (**7**)^18^, 2,3-dihydro-2–(4-hydroxy-3-methoxyphenyl)-5–(3-hydroxypropyl)-7-methoxy-3-benzofuranylmethyl *β*-d-xylopyranoside (**8**)^19^, protocatechuic acid (**9**)^20^, and 3,5,7-trihydroxy-2–(3,5-dihydroxyphenyl) chroman-4-one (**10**)^16^ (Figure 1). Compounds **4**, **8,** and **10** were isolated for the first time from this plant. Their structures were elucidated on the basis of 1 D and 2 D NMR analysis.

**Figure 2.**
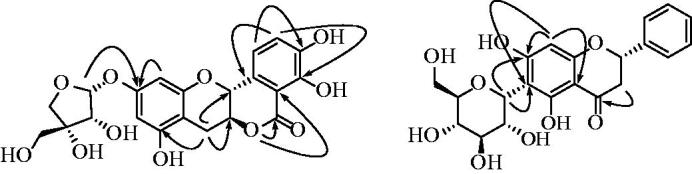
Key HMBC(**→**) correlations of compounds **1** and **2.**

Compound **1** was obtained as a brown amorphous powder, ${\left[\alpha \right]}_{D}^{22}$ −68.0° (MeOH, *c* 0.1), with UV absorption at 258 nm (log ε 6.08) and 334 nm (log ε 6.20). HR-ESI-MS in positive ion mode showed a molecular peak at *m/z* 471.0858 [M + Na]^+^, corresponding to C_21_H_20_O_11_. The ^1^H-NMR spectrum of compound **1** indicated the presence of two benzene moieties, as two doublet and two singlet signals. The ^13 ^C-NMR spectrum displayed signals for 21 carbons, including one carbonyl group at [δ 170.3 (C-5)], two methines bearing oxygen at [δ 77.3 (C-6a), 72.2 (C-12a)] and one methylene at [δ 27.2 (C-7)]. Compound **1** has a structure similar to compound **6**, but the HMBC spectrum confirmed that a carbonyl group was substituted on the B ring at [δ 108.1 (C-4a)]. This carbonyl group was linked to the hydroxyl group substituted on the C ring at [δ 77.3 (C-6a)] to make a D ring. The ^1^H-NMR data showed apiofuranoside moieties at [δ 5.49 (1H, d, *J* = 3.5 Hz, H-*1′*), 4.16 (1H, br s, H-*2′*), 4.09 (1H, d, *J* =  9.5 Hz, H-*4′*β), 3.87 (1H, d, *J* = 9.5 Hz, H-*4′*α), 3.63 (2H, br s, H-*5′*)], and the ^13 ^C-NMR data showed signals at [δ 108.8 (C-1′), 80.3 (C-3′), 78.3 (C-2′), 75.5 (C-4′), 64.9 (C-5′)] that were indicative of an apiofuranoside^10^. Additionally, the absolute stereochemistry of compound **1** was 6a*S* and 12a*R*, based on the coupling constants seen in the ^1^H-NMR data^21^. The linkage of this sugar at C-7 was established by HMBC. The key HMBC were as follows: H-1/C-3 and C-5; H-2/C-4 and C-12b; H-7/C-6a, C-8, and C-12; H-9/C-10; and H-11/C-10 (Figure 2 and Table 1). Thus, the structure of compound **1** was determined to be (6a*S*,12a*R*)-3,4,8-trihydroxy-6a-7-dihydroisochromeno[4,3-*b*]chromen-5(12a*H*)-one-10-*O*-*β*-apiofuranoside (**1**), which has not been reported previously in *U. davidiana*.

**Table 1.** ^1^H and ^13 ^C NMR data of compound **1** and **2** in CD_3_OD (600 MHz).

Compound 1			Compound 2		
	*δ_C_*	*δ_H_*		*δ_C_*	*δ_H_*
1	115.9	7.10 (1H, d, *J* = 8.1 Hz)	**1**		
2	123.0	7.16 (1H, d, *J* = 8.1 Hz)	**2**	80.4	4.95 (dd, *J* = 2.7, 12.5 Hz)
3	147.1		**3**	48.3	α = 3.12 (dd, *J* = 12.4, 17.1 Hz)
4	151.5				β = 2.82 (dd, *J* = 3.1, 17.1 Hz)
4a	108.1		**4**	197.7	
5	170.3		**5**	164.1	
6			**6**	106.2	
6a	77.3	4.62 (1H, td, *J*= 6.3, 10.6 Hz)	**7**	165.4	
7	27.2	α = 3.23 (1H, dd, *J* 6.3, 15.6 Hz)	**8**	96.5	6.0 (s)
		β = 2.80 (1H, dd, *J* = 10.6, 15.6 Hz)	**9**	164.4	
7a	102.2		**10**	103.1	
8	158.1		***1′***	129.7	
9	98.3	6.20 (1H, s)	***2′***	129.8	7.49 (d, *J* = 7.3 Hz)
10	158.8		***3′***	127.4	7.41 (t, *J* = 7.4 Hz)
11	97.0	6.22 (1H, s)	***4′***	140.4	7.36 (t, *J* = 7.2 Hz)
11a	155.9		***5′***	127.44	7.41 (t, *J* = 7.4 Hz)
12			***6′***	129.8	7.49 (d, *J* = 7.3 Hz)
12a	72.2	4.91 (1H, d, *J* = 10.8 Hz)	**1″**	75.2	4.79 (d, *J* = 9.9 Hz)
12b	130.8		**2″**	72.6	4.12 (m)
*1′*	108.8	5.49 (1H, d, *J* = 3.5 Hz)	**3″**	80.2	3.40 (m)
*2′*	78.3	4.16 (1H, br s)	**4″**	71.8	3.40 (m)
*3′*	80.3		**5″**	82.5	3.40 (m)
*4′*	75.5	α = 3.87 (1H, d, *J* = 9.5 Hz)	**6″**	62.9	α = 3.86 (dd, *J* = 1.9, 12.0 Hz)
		β = 4.09 (1H, d, *J* = 9.5 Hz)			β = 3.71 (dd, *J* = 5.5, 12.0 Hz)
*5′*	64.9	3.63 (2H, br s)			

Compound **2** was obtained as a brown amorphous powder, ${\left[\alpha \right]}_{D}^{22}$ +56.0˚ (MeOH, *c* 0.001), with ultraviolet (UV) absorption at 290 nm (log ε 6.11). HR-ESI-MS in positive ion mode showed a molecular peak at *m/z* 441.1151 [M + Na]^+^, calculated as C_21_H_22_O_9_. We found a close structural relationship between compounds **2** and **3**, reflected in their similar spectral features. The most significant difference between the ^1^H and ^13 ^C-NMR spectra of compounds **2** and **3** was an aromatic B ring. The ^1^H-NMR spectrum of compound **2** revealed the presence of a mono-substituted benzene moiety as one doublet signal at [δ 7.49 (2H, d, *J*= 7.3 Hz, H-*2′*, H-*6′*)] and two triplets at [δ 7.41 (2H, t, *J*= 7.4 Hz, H-*3′*, H-*5′*)], [δ 7.36 (1H, t, *J*= 7.2 Hz, H-*4′*)]. The ^13 ^C-NMR spectrum of compound 1 showed peaks at [δ 127.4 (C-*3′*, C-*5′*), 140.4 (C-*4′*)]. The ^1^H-NMR spectrum showed one C-glucose moiety, with its anomeric proton signal at [δ 4.79 (1H, d, *J* = 9.9 Hz, H-*1′'*)] and the corresponding ^13 ^C-NMR carbon signal at [δ 75.2 (C-*1′'*)], in the characteristic regions of C-substituted glucoside. The coupling constant of the signal resulting from the anomeric proton of the glucopyranoside indicates a *β*-configuration of the glycosidic linkage [4.79 (1H, d, *J*= 9.9 Hz, H-*1′'*)]. Further, the position of the glucosyl moiety in compound **2** at C-*1′'* was confirmed by heteronuclear multiple bond correlation (HMBC) of the anomeric proton to C-6 and C-7. The key HMBCs were as follows: H-8/C-6, C-7 and C-10 at the A-ring; and H-3/C-4 at the C-ring (Figure 2 and Table 1). Finally, the absolute configuration at C-2 was determined to be *S* compared to the similar structure of artocarpin F, according to the circular dichroism (CD) spectroscopic analysis, which showed negative and positive Cotton effects at 290 and 334 nm, respectively^22^. Thus, considering these spectral data, we determined compound **2** to be pinocembrin 6-*C*-β-D-glucoside^23^.

### Inhibitory effects of compounds on sEH

Some studies have been conducted to develop new sEH inhibitors derived from natural plants^7^^,^^24–26^. Several natural products containing flavonoid and benzofuran moieties have been found to have inhibitory activity against sEH^7^^,^^24^. Our efforts led to the isolation and identification of compounds with similar scaffolds to those mentioned above.

We performed methanol extraction on the root bark of *U. davidiana*, and isolated 10 compounds (**1**–**10**) to evaluate the inhibitory activity against catalytic sEH *in vitro* using AUDA (IC_50_ value = 2.0 ± 0.2 nM) as a positive control (Equation (1)). Compounds **1**, **2**, **4**, **6**–**8**, and **10** had an inhibitory rate over 50%, while for compounds **3**, **5**, and **9** the rate was under 38%. As indicated in Table 2, seven inhibitors (**1**, **2**, **4**, **6**–**8**, and **10**) had IC_50_ values ranging from 11.4 ± 2.3 to 36.9 ± 2.6 µM by Equation (2). Of interest, the two novel compounds **1** and **2** demonstrated acceptable inhibitory activity of 14.5 ± 0.5 and 11.4 ± 2.3 µM, respectively.

**Table 2.** sEH inhibitory effect of isolated compounds **1**–**10**.

Compound	Inhibition of compounds on sEH^a^	
	100 μM (%)	IC_50_ (μM)
**1**	59.4 ± 2.5	14.5 ± 0.5
**2**	91.8 ± 4.5	11.4 ± 2.3
**3**	21.7 ± 2.5	N.T
**4**	65.9 ± 0.8	26.3 ± 4.5
**5**	26.1 ± 4.0	N.T
**6**	69.8 ± 2.2	16.0 ± 3.2
**7**	55.6 ± 0.7	23.0 ± 0.7
**8**	64.8 ± 1.4	36.9 ± 2.6
**9**	37.3 ± 1.9	N.T
**10**	70.7 ± 0.4	16.1 ± 3.2
AUDA^b^	2.0 ± 0.2 nM	

^a^ Compounds were tested three times.

^b^ Positive control.

### Molecular docking

**Figure 3.**
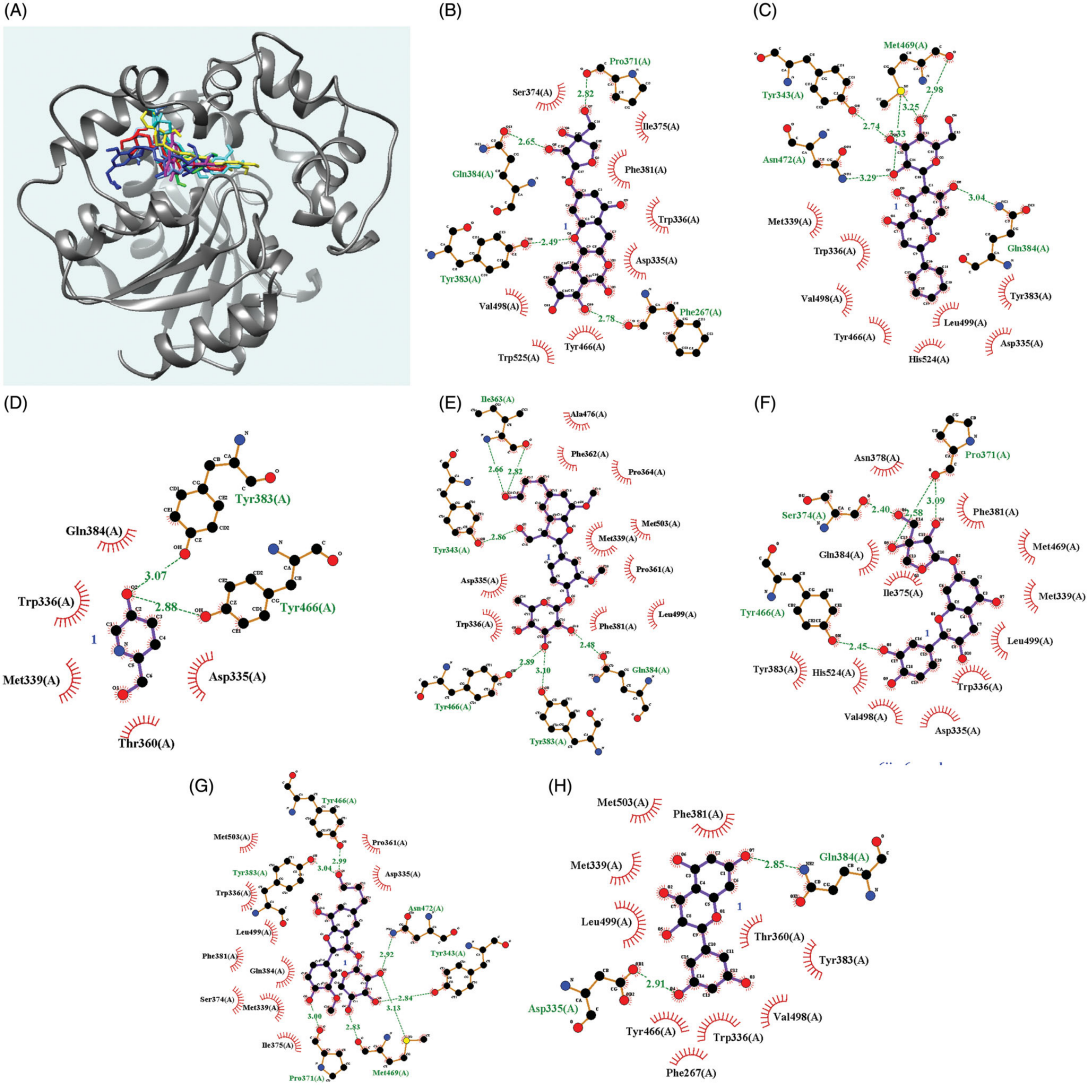
The best pose (**A**) and the hydrogen-bond interactions of the active site with ligands **1**, **2**, **4**, **6**–**8** and **10** (**B**–**H**), respectively.

Next, we simulated the interaction force between sEH and each potential inhibitor using molecular docking, with a grid that mapped the activity site of sEH. As indicated in Table 3, seven inhibitors had low AutoDock (range: −4.23 to −9.51 kcal/mol). Inhibitors **1** and **2** (for compounds **1** and **2**) maintained six hydrogen bonds (Tyr343 [2.74 Å], Gln384 [3.04 Å], Met469 [2.98 Å, 3.25 Å, and 3.33 Å] and Asn472 [3.29 Å]) and four hydrogen bonds (Phe267 [2.78 Å], Pro371 [2.82 Å], Tyr383 [2.49 Å], and Gln384 [2.65 Å]). Inhibitor **4** had bonds with Tyr383 (3.07 Å) and Tyr466 (2.88 Å). Inhibitor **6** had six hydrogen bonds (Tyr383 [3.10 Å], Gln384 [2.48 Å], Tyr466 [2.89 Å], Tyr343 [2.86 Å], and Ile363 [2.66 Å and 2.82 Å]) of sEH. Inhibitor **7** interacted with four amino acids (Pro371 [3.09 Å and 2.58 Å], Ser374 [2.40 Å], and Tyr466 [2.45 Å]). Inhibitor **8** contained seven hydrogen bonds with six amino acids (Tyr343 [2.84 Å], Pro371 [3.00 Å], Tyr383 [3.04 Å], Tyr466 [2.99 Å], Met469 [2.83 Å and 3.13 Å], and Asn472 [2.92 Å]). Finally, inhibitor **10** interacted with two amino acids (Asp335 [2.91 Å] and Gln384 [2.85 Å]) (Figure 3 and Table 3).

**Table 3.** Interaction of inhibitor and autodock score for sEH.

	Autodock score (kcal/mol)	Hydrogen bonds (Å)
**1**	−8.54	Phe267(2.78), Pro371(2.82), Tyr383(2.49), Gln384(2.65)
**2**	−8.34	Tyr343(2.74), Gln384(3.04), Met469(2.98,3.25,3.33), Asn472(3.29)
**4**	−4.23	Tyr383(3.07),Tyr466(2.88)
**6**	−7.95	Tyr383(3.10), Gln384(2.48), Tyr466(2.89), Tyr343(2.86), Ile363(2.66,2.82)
**7**	−8.85	Pro371(3.09,2.58), Ser374(2.40), Tyr466(2.45)
**8**	−9.51	Tyr343(2.84), Pro371(3.00), Tyr383(3.04), Tyr466(2.99), Met469(2.83,3.13), Asn472(2.92)
**10**	−6.75	Asp335(2.91), Gln384(2.85)

### Molecular dynamics

**Figure 4.**
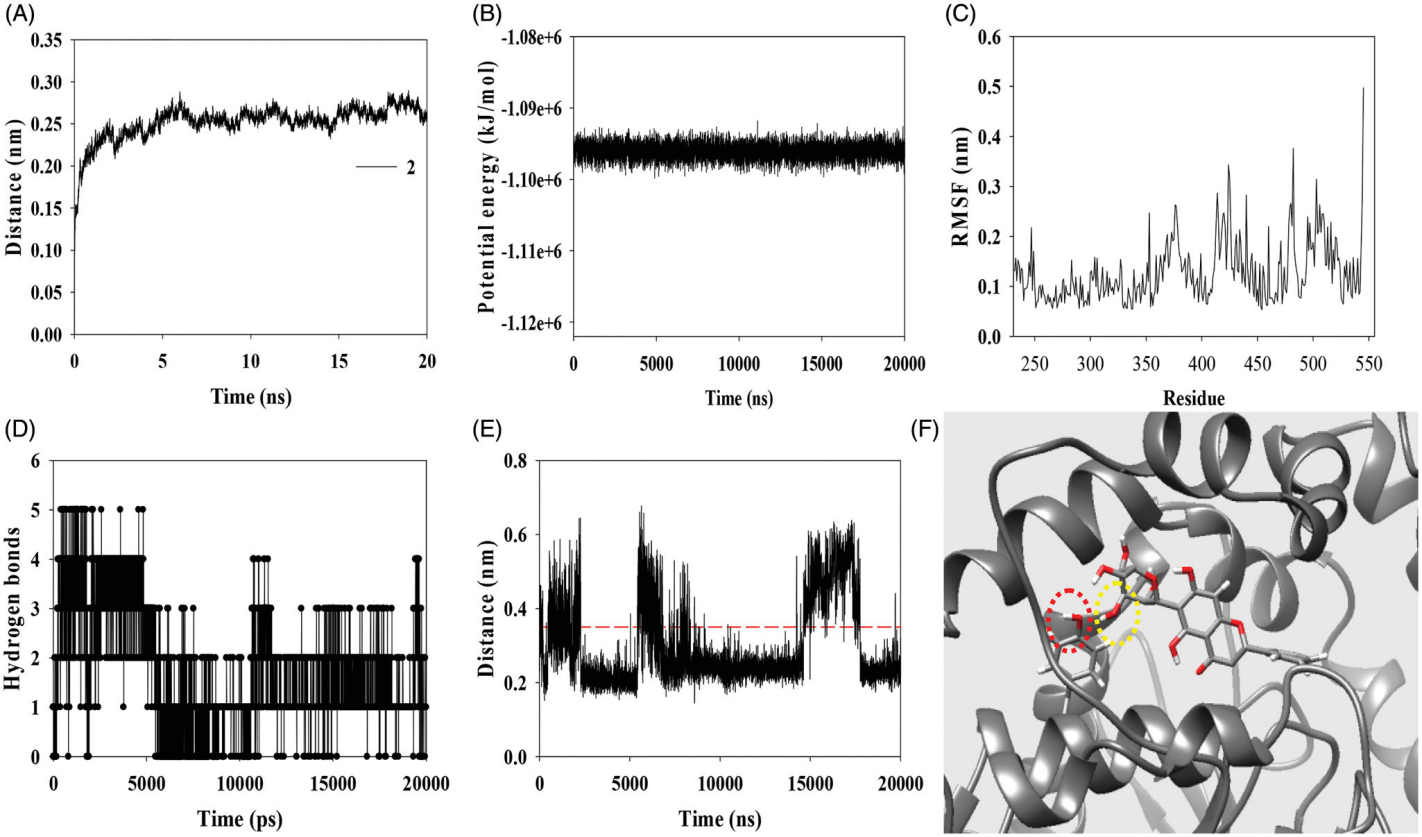
The RMSD (A), the potential energy (B), RMSF (C), and hydrogen bonds (D) of simulated interaction between sEH and inhibitor **2** calculated during 20 ns. The distance of 3″ hydroxyl group of inhibitor **2** from hydroxyl group of Tyr343 (E, F).

MD is the state-of-the-art research technology for the development of targeted enzyme inhibitors along with molecular docking^27^. Our MD was a study that calculated the interaction of flexible enzyme with flexible inhibitor under 300 K temperature and 1 bar pressure in water solvent containing 6 Cl anions. The rigid complex between sEH and inhibitor **2** of the docking was put into a relaxed state by energy minimisation, NVT, and NPT in Gromacs 4.6.5., respectively. The corresponding product was simulated MD for 20 ns. As showed in Figure 4(A,B), the root mean square deviation (RMSD) values were stably under 3 Å with the potential energy of approximately −1.095 × 10^6^ kJ/mol for simulation trajectory. The enzyme residues affected by inhibitor **2** showed fluidity under 4 Å of the root mean-square fluctuations (RMSF) values (Figure 4(C)). It was confirmed that their complex maintained 0–5 hydrogen bonds over time (Figure 4(D)). The hydrogen bonds between inhibitor **2** and sEH residues were analysed at 2 ns intervals (Table S1). It was showed that glucose group of **2** was constantly made hydrogen bonds with Tyr343 residue. As indicated in Figure 4(E, F), inhibitor was continuously approached by the distance within 3 Å to this amino acid except for mainly ∼15 to ∼17.5 ns during the 20 ns simulation time. In particular, molecular docking result revealed that four amino acids (Tyr343, Gln384, Met469, and Asn472) are important residues for hydrogen bonds. Furthermore, MD, an in-depth computational simulation study, found that Tyr343 is the most important residue for binding the inhibitor. In molecular docking, inhibitor can induce forced bonding by docking to a rigid enzyme. On the other hand, MD is the skill to find the bond between the inhibitor and the amino residue in a fluid state based on molecular force. Therefore, through sequential experiments, it was possible to find amino residue (Tyr343) that participates in hydrogen bonding with a high probability for inhibitor.

## Conclusion

Among compounds **1**–**10** isolated from the root back of *U. davidiana*, a new compound **1** and compound **2** were purified for the first time from natural plants, and known compounds **4**, **8,** and **10**, were isolated for the first time from this plant. Seven compounds (**1**, **2**, **4**, **6**–**8**, and **10**) had IC_50_ values under 37 µM on sEH. Two compounds **1** and **2** were confirmed to be potential inhibitors of sEH, with IC_50_ values of 11.4 ± 2.3 and 14.5 ± 0.5 µM, respectively. Additionally, molecular docking was used to describe the binding of each inhibitor with sEH. The complex of sEH with the potential inhibitor **2** was shown to be stable, as indicated by the low binding energy calculated by autodocking. Additionally, MD study proved that glucose group of inhibitor **2** was interacted with hydroxyl group of Tyr343 as key amino acid within 3 Å distance. Finally, these findings suggest that inhibitor **2** may help as a lead compound in the development of new cardiovascular disease treatments, and as a prescription enhancer along with typical urea and amide-based sEH inhibitors.

## Supplementary Material

Supplemental Material
