# Supplementary material for: Inhibition of soluble epoxide hydrolase by phytochemical constituents of the root bark of Ulmus davidiana var. japonica
Source: J Enzyme Inhib Med Chem. 2021 May 17;36(1):1049–55. doi: 10.1080/14756366.2021.1927005 (PMC8153708; doi:10.1080/14756366.2021.1927005)
Supplement: Supplemental Material [file IENZ_A_1927005_SM2837.pdf]

Inhibition of soluble epoxide hydrolase by phytochemical constituents of the root bark of *Ulmus davidiana var. japonica*

Jang Hoon Kim[a,*], Ji Su Park[b,*], Yun Ji Lee[a], Sena Choi[a], Young Ho Kim[b], Seo Young Yang[b,c**]

[a]*Department of Herbal Crop Research, National Institute of Horticultural & Herbal Science, RDA, Eumsung 27709, Korea;*

[b]*College of Pharmacy, Chungnam National University, Daejeon 34134, Republic of Korea*

[c]*Department of Pharmaceutical Engineering, Sangji University, 83 Sangidae-gil, Wonju-si, Gangwon-do 26339, Republic of Korea*

* These authors contributed equally to this work.

**Corresponding authors

Dr. Seo Young Yang

Tel., +82-42-821-5933, Fax, +82-42-823-6566; E-mail address, syyang@cnu.ac.kr/ syyang317@gmail.com/ syyang@sangji.ac.kr (Y.S. Yang)

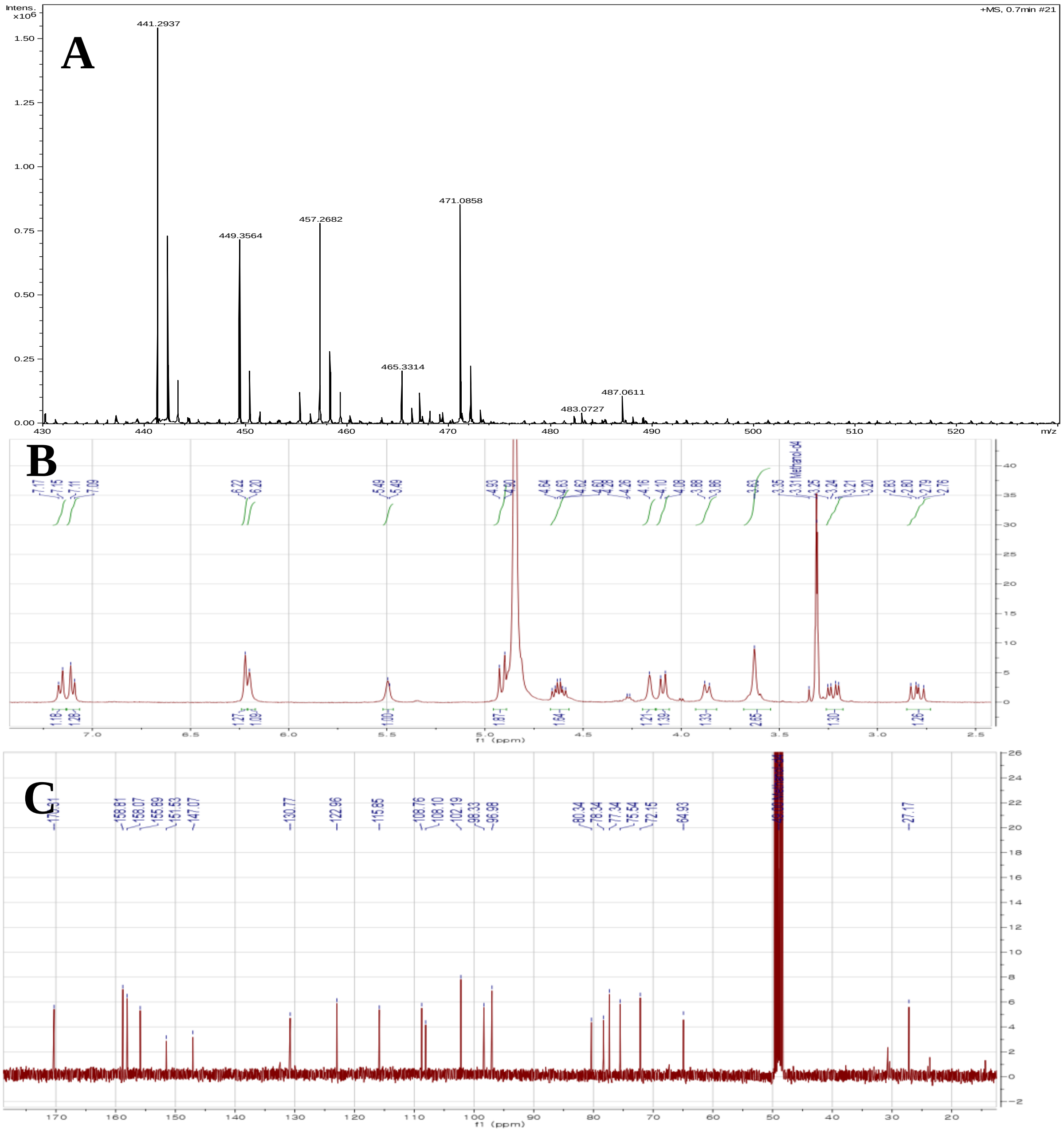

Figure S1. HRESIMS (A), ^{1}H NMR (B), and ^{13}C NMR (C) spectra of compound **1** (^{1}H NMR: 600 MHz, ^{13}C NMR: 150 MHz, MeOD-d_4).

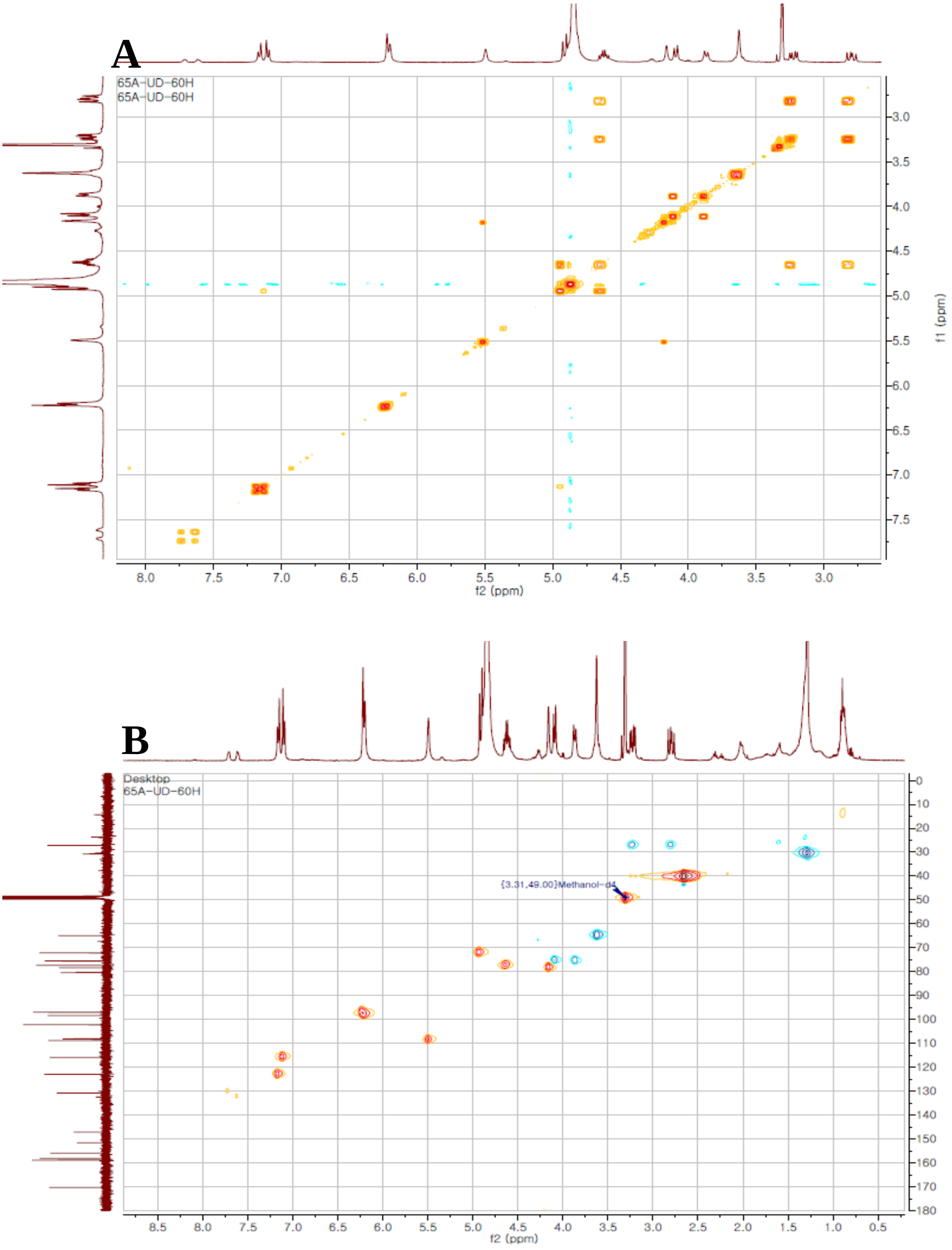

Figure S2. COSY (A) and HMQC (B) of compound **1**.

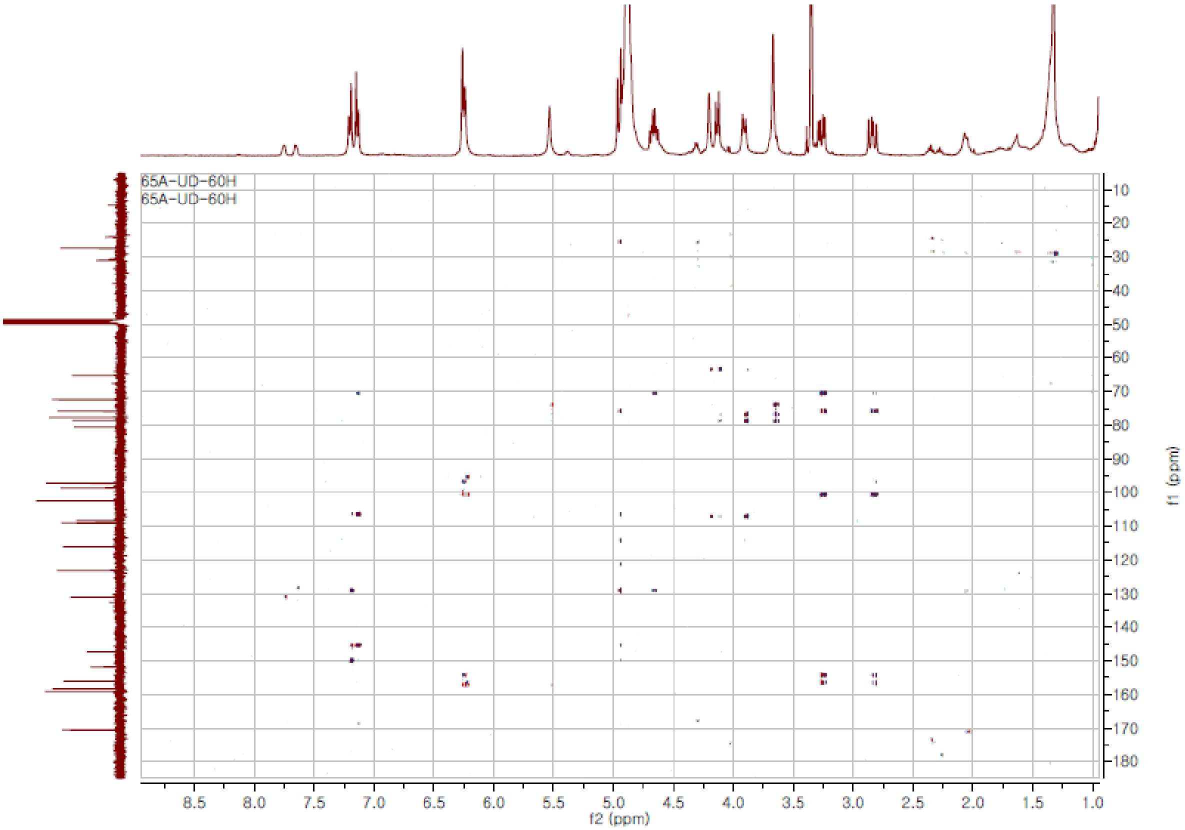

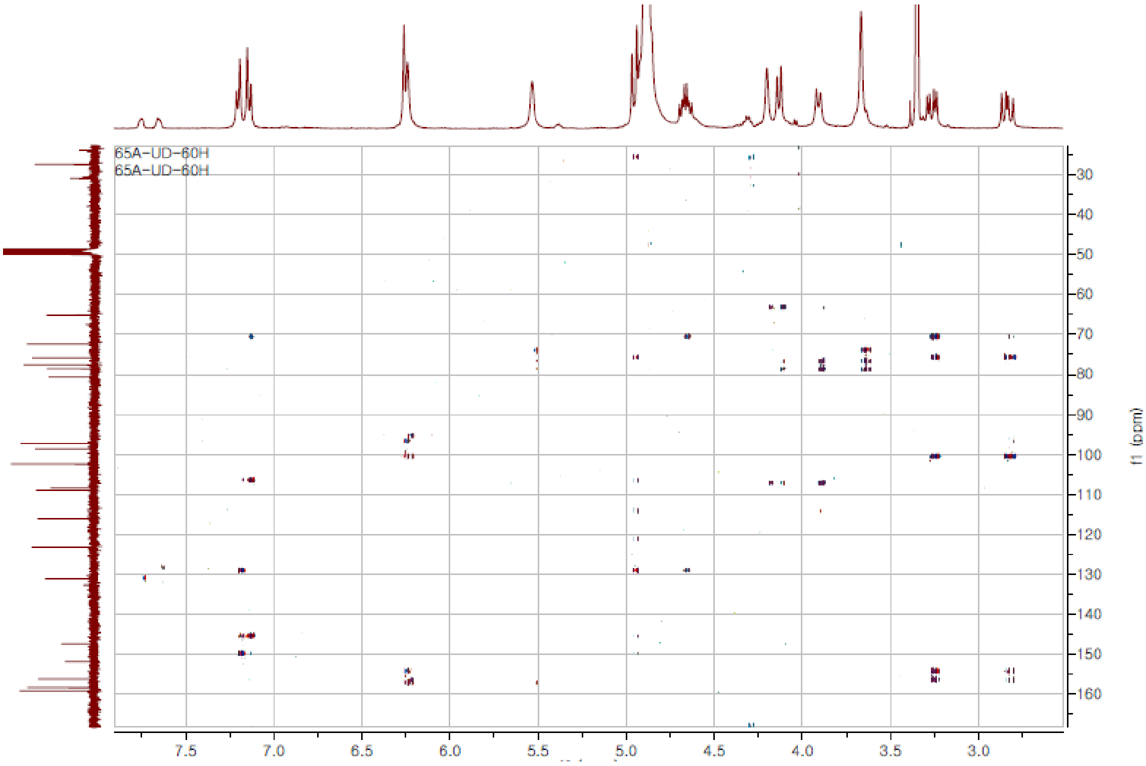

Figure S3. HMBC spectrum of compound **1**.

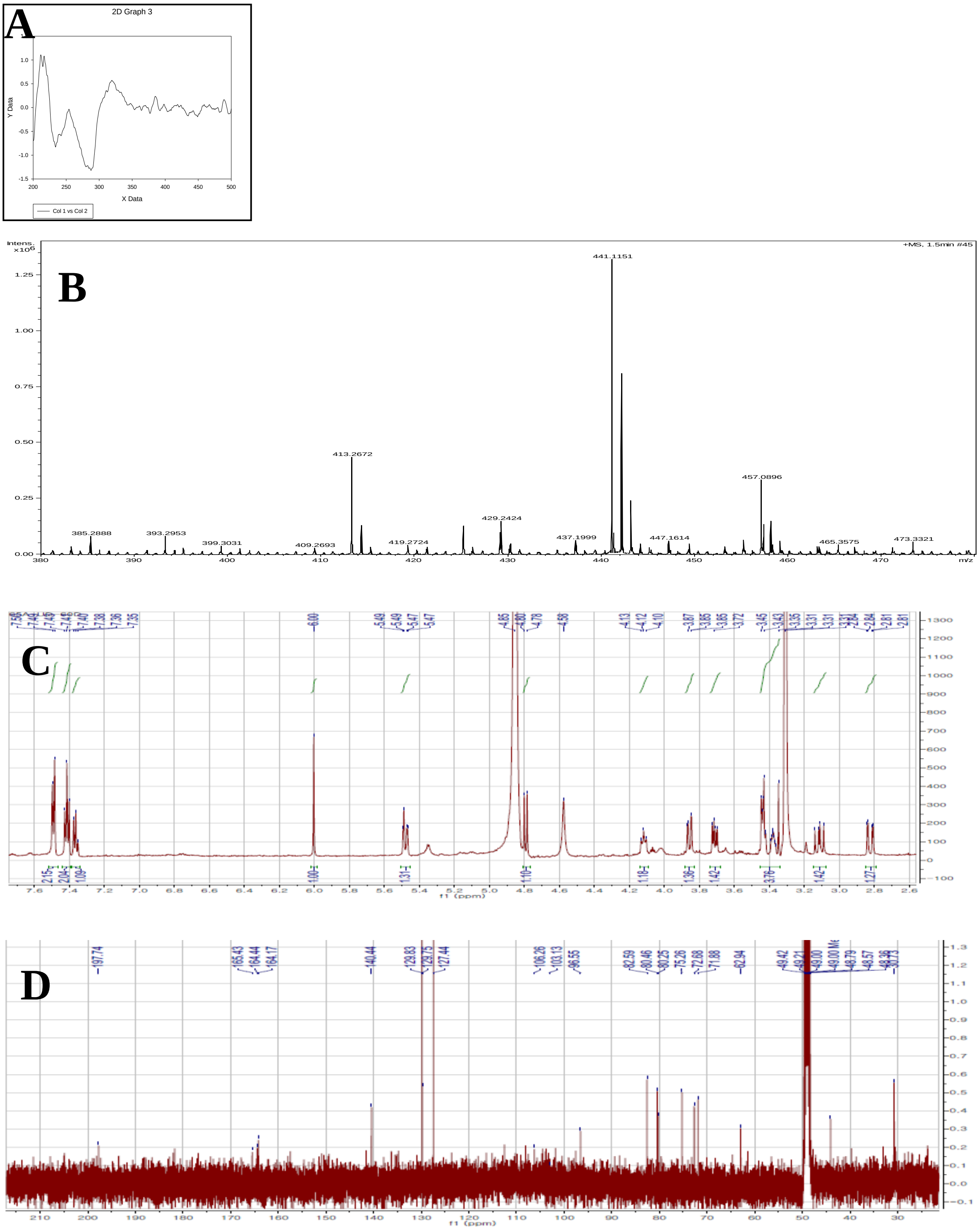

Figure S4. CD (A), HRESIMS (B), ^{1}H NMR (C), and ^{13}C NMR (D) spectra of compound **2**. (^{1}H NMR: 600 MHz, ^{13}C NMR: 150 MHz, MeOD-d_4).

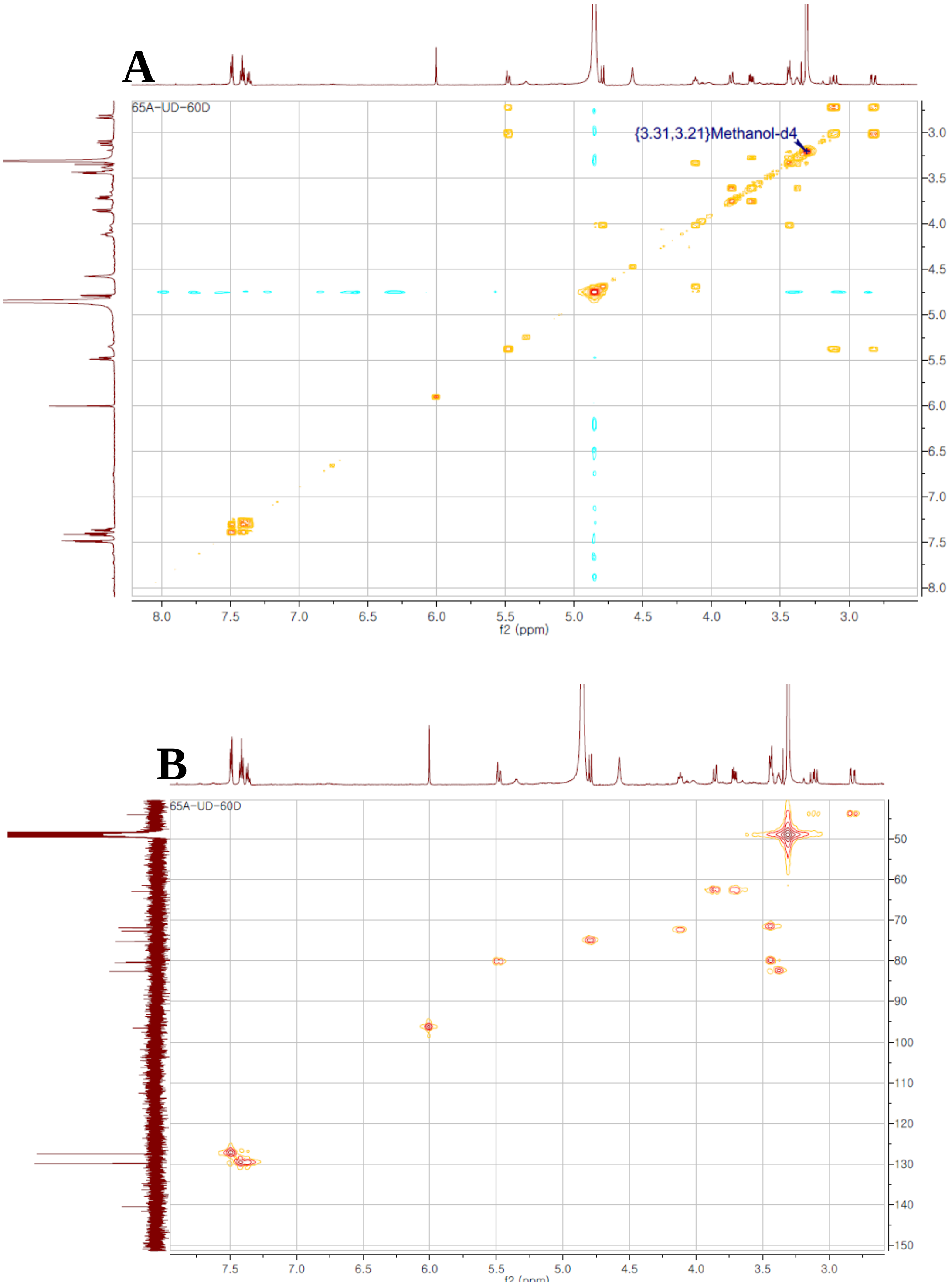

Figure S5. COSY (A) and HMQC (B) spectra of compound **2**.

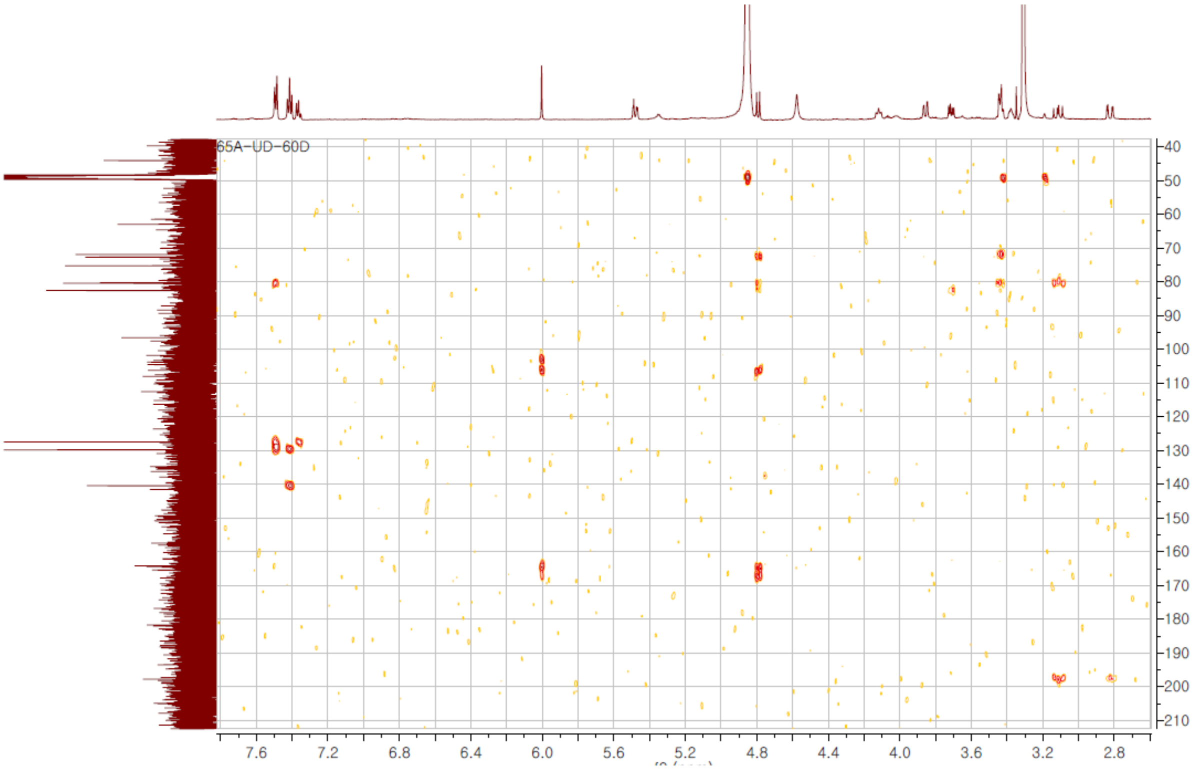

Figure S6. HMBC spectrum of compound **2**.

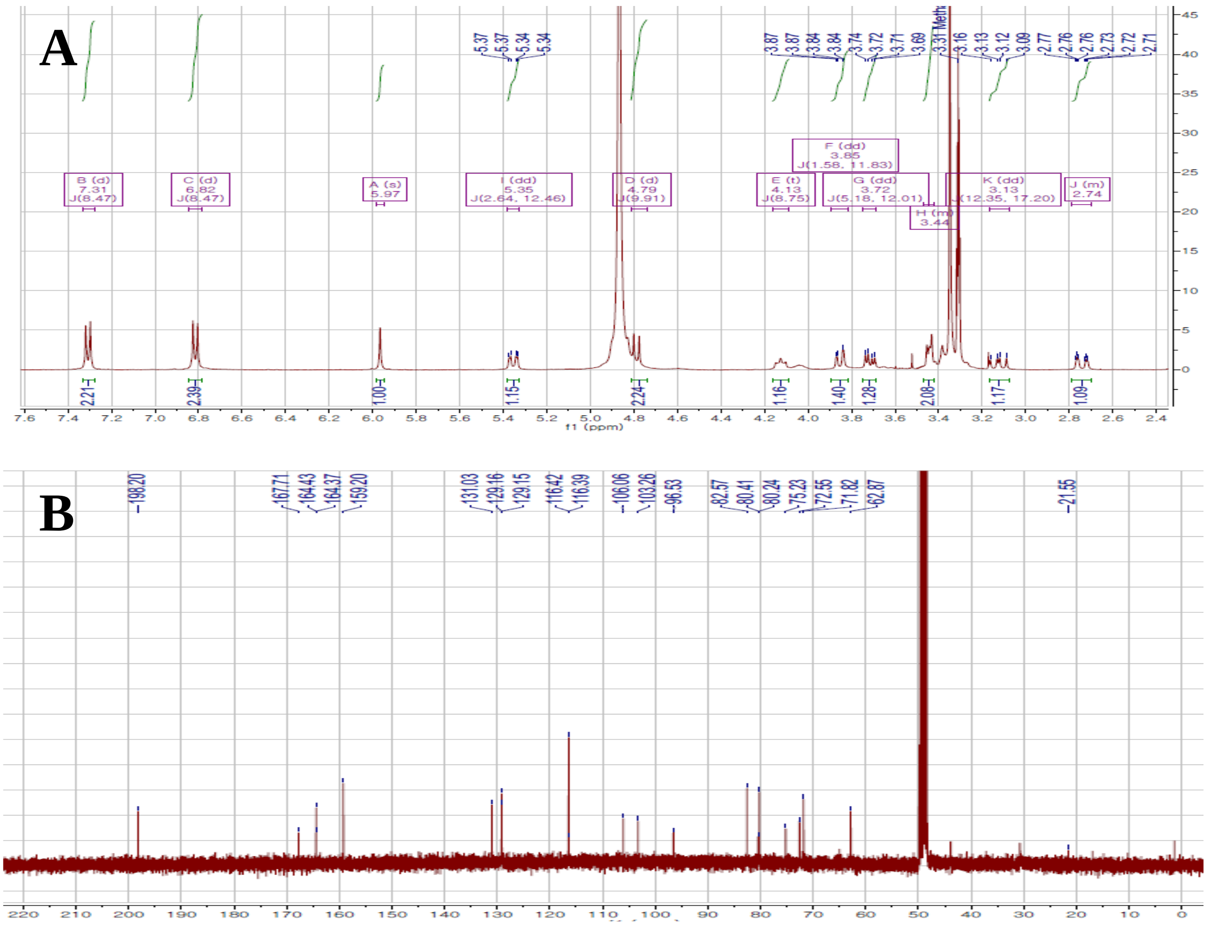

Figure S7. ^{1}H NMR (A) and ^{13}C NMR (B) spectra of compound **3** (^{1}H NMR: 300 MHz, ^{13}C NMR: 85 MHz, MeOD-d_4).

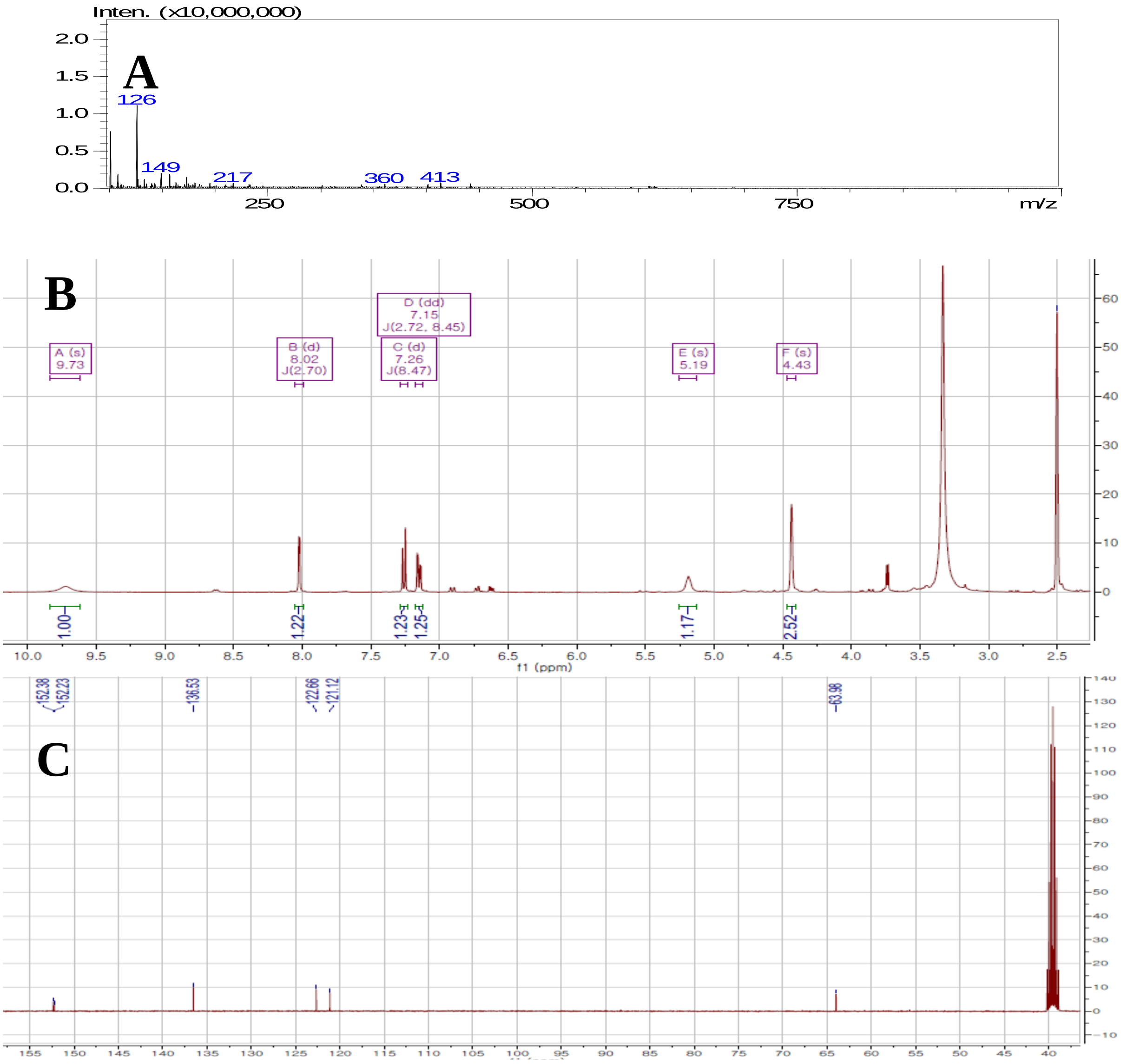

Figure S8. MS (A), ^{1}H NMR (B), and ^{13}C NMR (C) spectra of compound **4** (^{1}H NMR: 300 MHz, ^{13}C NMR: 85 MHz, DMSO-d_6).

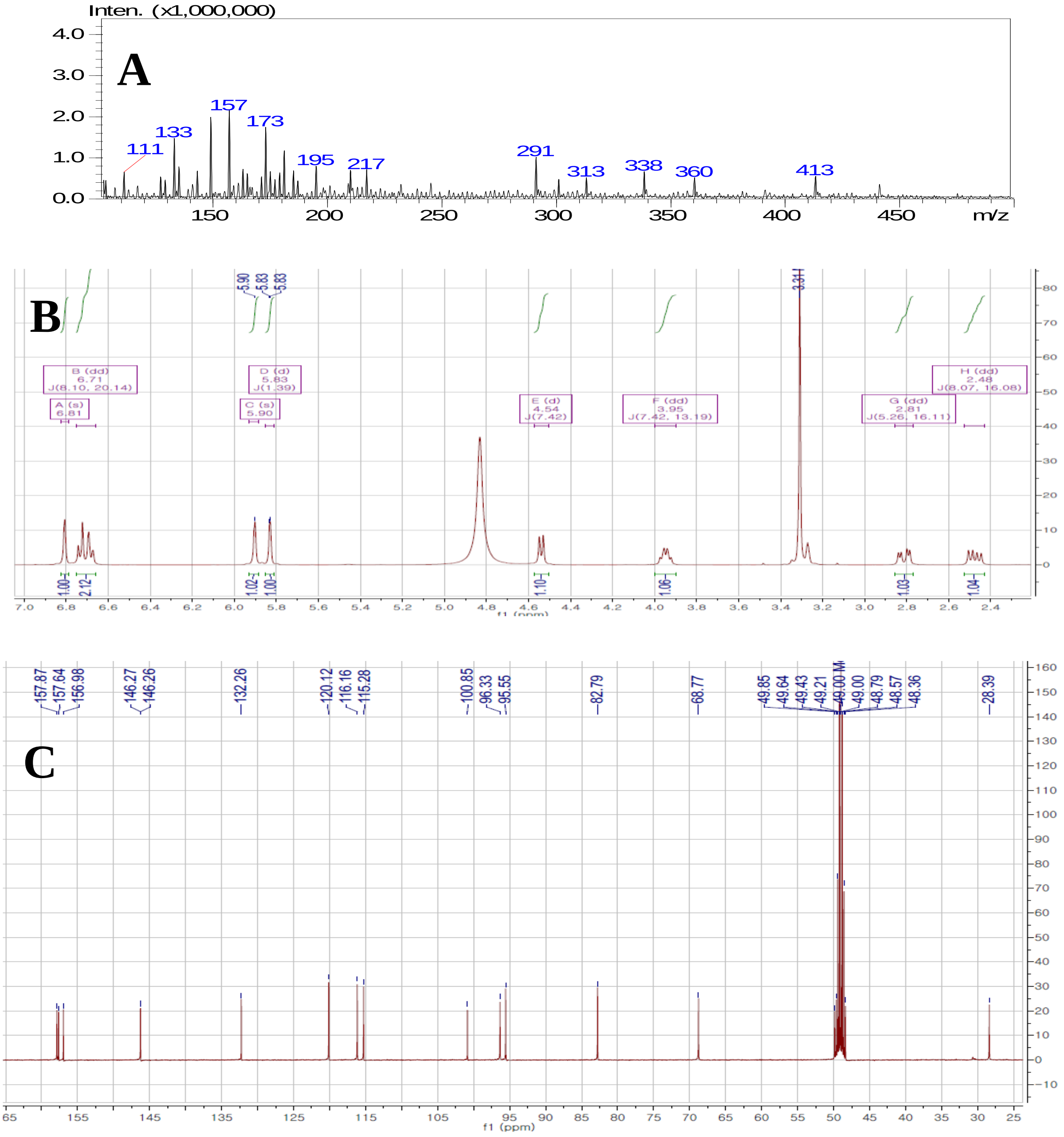

Figure S9. MS (A), ^{1}H NMR (B), and ^{13}C NMR (C) spectra of compound **5** (^{1}H NMR: 300 MHz, ^{13}C NMR: 85 MHz, MeOD-*d*$_4$).

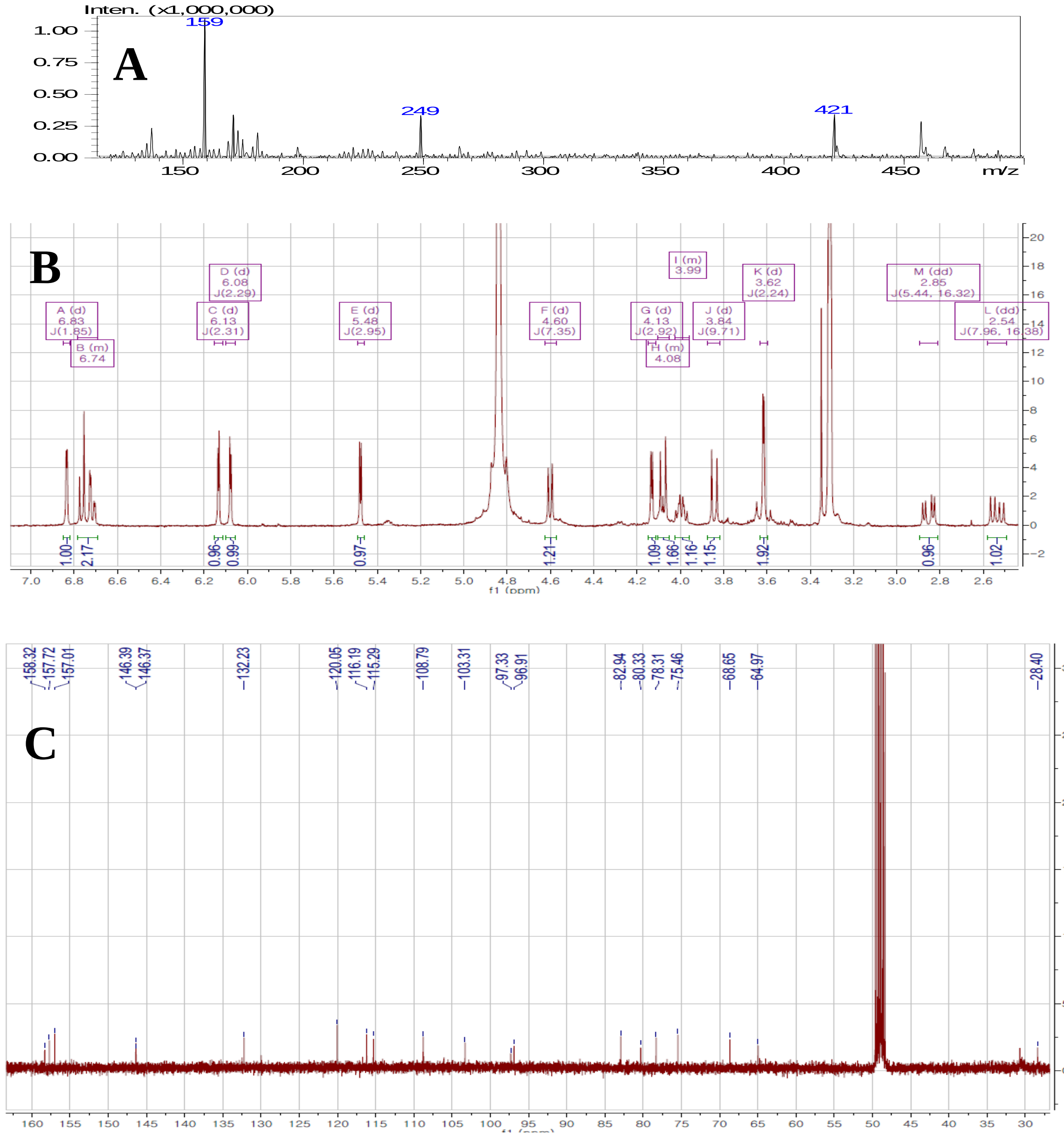

Figure S10. HRESIMS (A), ^{1}H NMR (B), and ^{13}C NMR (C) spectra of compound **6** (^{1}H NMR: 300 MHz, ^{13}C NMR: 85 MHz, MeOD-d_4).

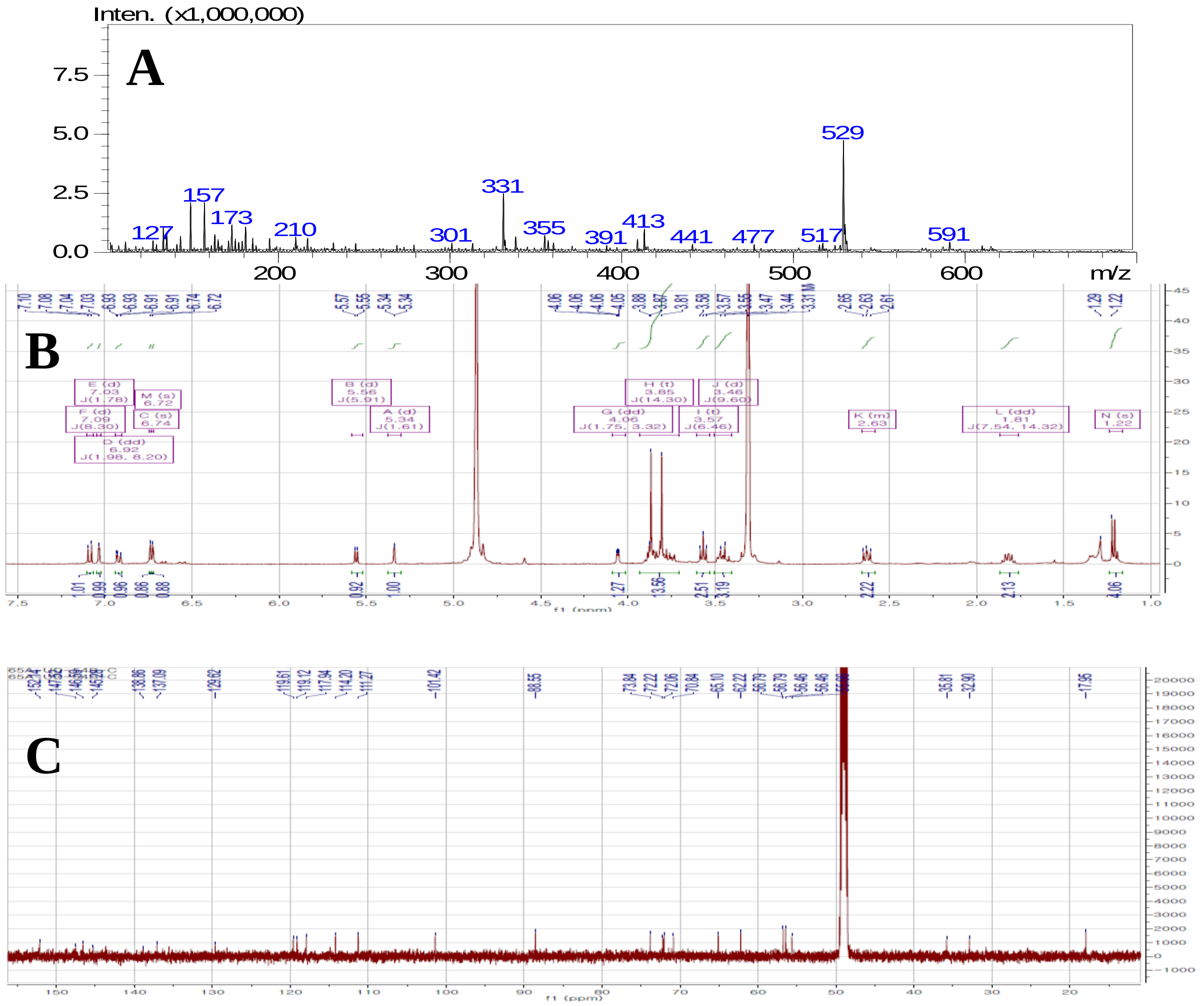

Figure S11. HRESIMS (A), ^{1}H NMR (B), and ^{13}C NMR (C) spectra of compound **7** (^{1}H NMR: 300 MHz, ^{13}C NMR: 85 MHz, MeOD-d_4).

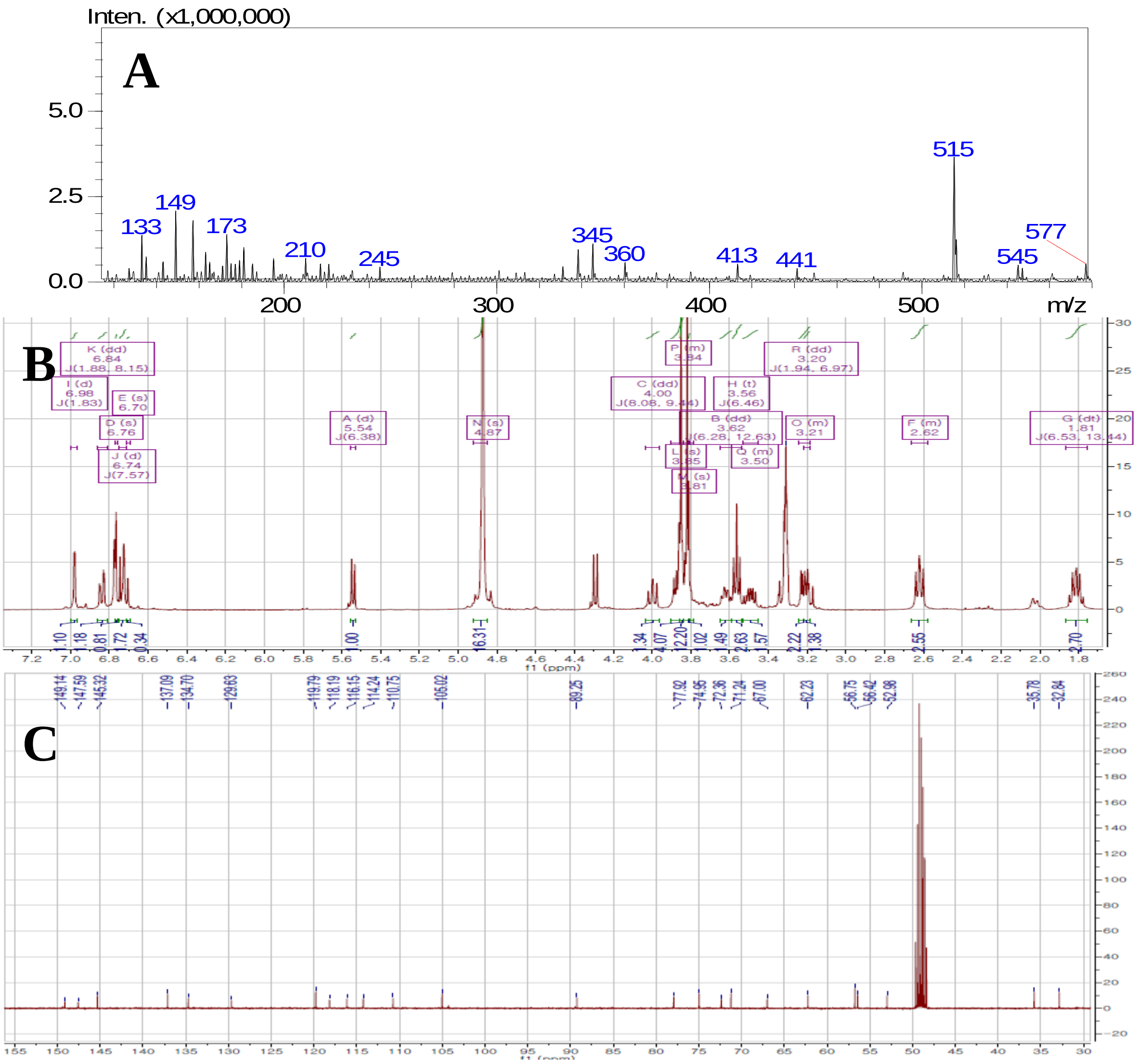

Figure S12. HRESIMS (A), ^{1}H NMR (B), and ^{13}C NMR (C) spectra of compound **8** (^{1}H NMR: 300 MHz, ^{13}C NMR: 85 MHz, MeOD-d_4).

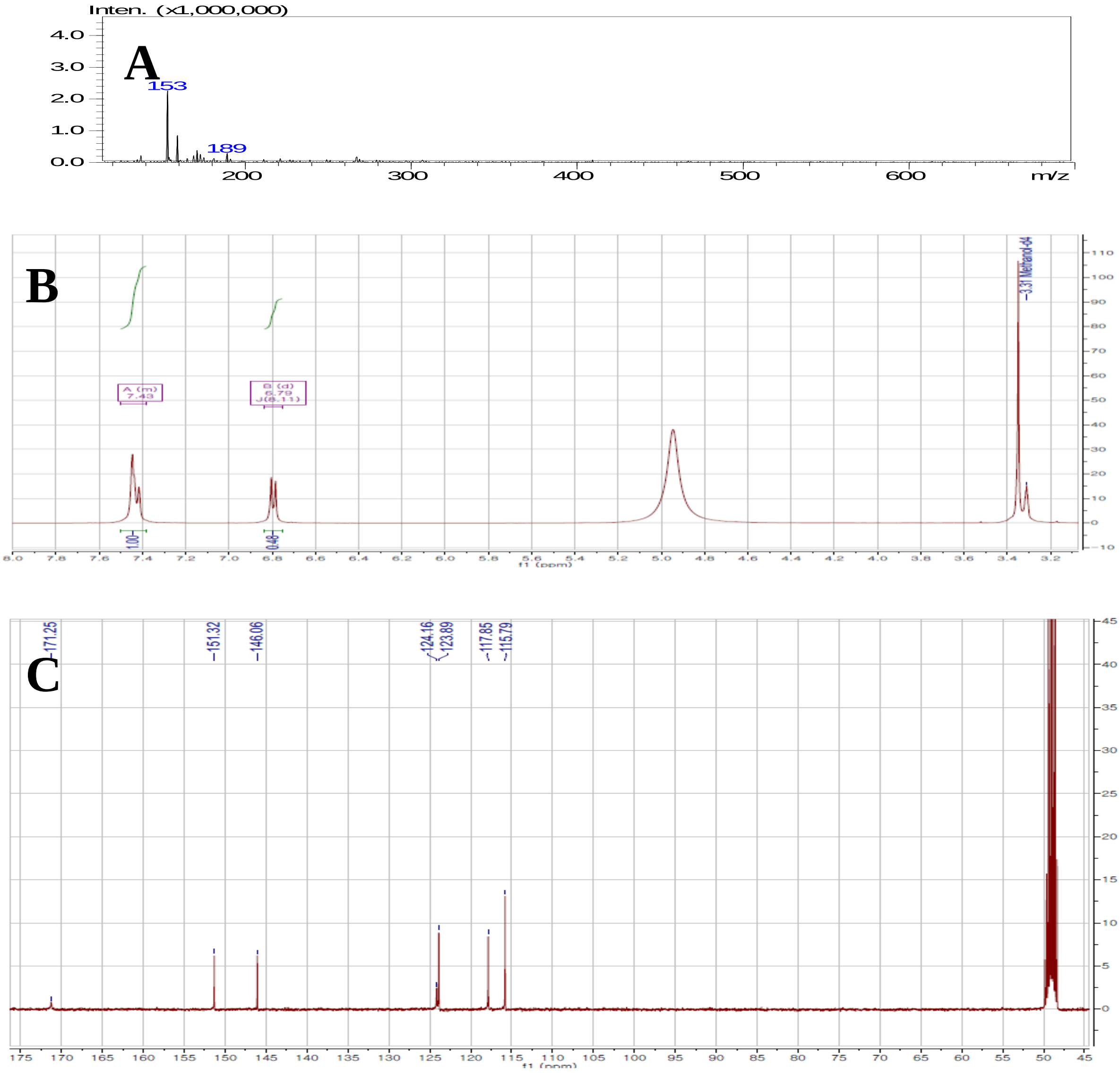

Figure S13. MS (A), ^{1}H NMR (B), and ^{13}C NMR (C) spectra of compound **9** (^{1}H NMR: 300 MHz, ^{13}C NMR: 85 MHz, MeOD-d_4).

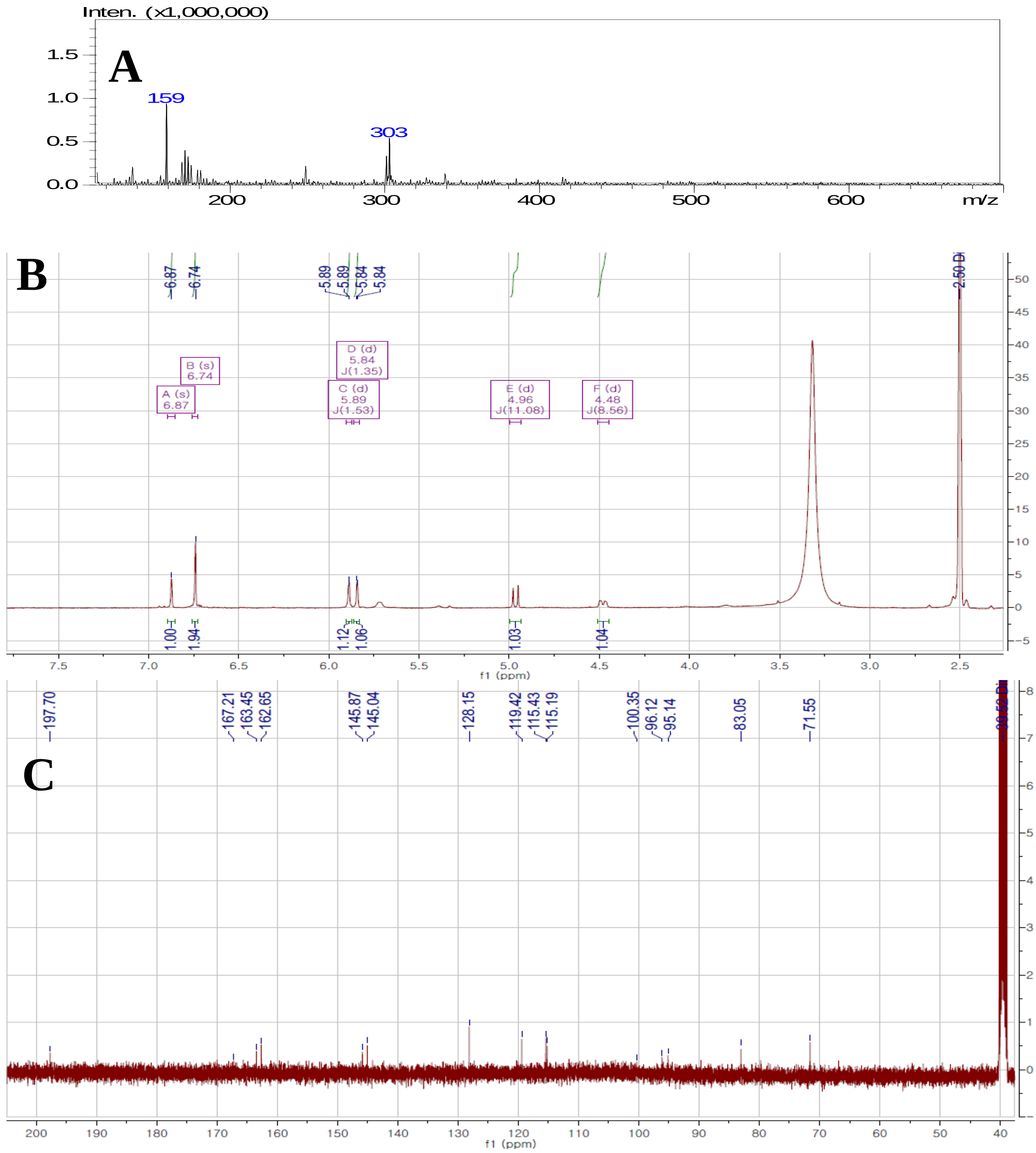

Figure S14. MS (A), ^{1}H NMR (B), and ^{13}C NMR (C) spectra of compound **10** (^{1}H NMR: 300 MHz, ^{13}C NMR: 85 MHz, DMSO-d_6).

Table S1. Hydrogen bonds analysis of inhibitor **2** with sEH at 2 ns intervals for simulation.

Time	Hydrogen bonds
**0**	Tyr343(2.74), Met469(3.17)
**2**	Gln384(2.68), Asn472(3.16)
**4**	Tyr343(2.62), Ile363(2.68), Gln384(2.64), Asn472(3.24)
**6**	Gln384(3.22)
**8**	Tyr343(2.94)
**10**	Tyr343(2.86)
**12**	Tyr343(2.87), Met238(2.89)
**14**	Met339(3.34), Tyr343(2.83), Gln384(3.04)
**16**	Tyr343(2.99), Pro364(3.29)
**18**	Tyr343(2.60), Gln388(3.27)
**20**	Tyr343(2.54)
